# Stability Switches of Arbitrary High-Order Consensus in Multiagent Networks with Time Delays

**DOI:** 10.1155/2013/514823

**Published:** 2013-09-11

**Authors:** Bo Yang

**Affiliations:** School of Automation, Wuhan University of Technology, Wuhan 430070, China

## Abstract

High-order consensus seeking, in which individual high-order dynamic agents share a consistent view of the objectives and the world in a distributed manner, finds its potential broad applications in the field of cooperative control. This paper presents stability switches analysis of arbitrary high-order consensus in multiagent networks with time delays. By employing a frequency domain method, we explicitly derive analytical equations that clarify a rigorous connection between the stability of general high-order consensus and the system parameters such as the network topology, communication time-delays, and feedback gains. Particularly, our results provide a general and a fairly precise notion of how increasing communication time-delay causes the stability switches of consensus. Furthermore, under communication constraints, the stability and robustness problems of consensus algorithms up to third order are discussed in details to illustrate our central results. Numerical examples and simulation results for fourth-order consensus are provided to demonstrate the effectiveness of our theoretical results.

## 1. Introduction

Our understanding of distributed cooperative control for both natural and engineered dynamic networks has been advanced in the last few years [1–8]. Particularly, it is found that the research on shared information of interest in the network of dynamic agents facilitates significantly the distributed coordinated control [8–16]. Therefore, an essential issue for successful coordination is to design effective algorithms so that the agents in the network can converge to a consistent sense or view of the shared information of interest. A consensus algorithm is an interaction rule that governs the information exchange between a dynamic agent and all of its neighbors in the network. Notwithstanding original studies in the area of computer science (particularly in distributed computation and automata), the consensus problems discussed previously have been studied extensively in the context of distributed coordination of dynamic networks, partly due to the potential applications including congestion control in communication networks, cooperative control of multiple vehicle systems, formation control, swarming and flocking, distributed sensor network, attitude alignment of groups of satellites, air traffic control, and many others [17–20]. 

It is noticed that most algorithms focus on the consensus problems for the networks of agents with single or double integrator dynamics. In the current paper, we investigate arbitrary high-order consensus networks in presence of communication time-delays, which generalize the aforementioned existing results in the literature. The idea of employing high-order integrator dynamic agents under communication constraints is inspired by the following reasons. 

First, from the point of view of system science, nonlinear systems of a broad class (i.e., feedback linearizable systems) can be transformed to linear systems via feedback control and transformation of the state vector by using differential-geometric methods [21]. Hence, if there exist algorithms solving the consensus problems for networks of agents with dynamics described by the high-order integrator, then consensus controllers can be readily designed for nonlinear dynamic networks. Second, observing flocking, schooling, and swarming behaviors in nature has truly inspired that biological networks may also have to build consensus on acceleration or even jerk to maintain the collective behaviours in some sudden events (for instance, when one of a fish school is suddenly aware of some source of foods or threats) [22]. Third, due to the limited communication capacity of dynamic agents, the communication time-delays are inevitable in reality. It is well known that unmodeled delay effects may deteriorate the performance of the system and even destabilize it [23]. Therefore, it is of both theoretical and practical interest to pursue the consensus problems in our general framework. 

In our earlier work [16], it was shown that the forced second-order consensus with delayed input can be achieved asymptotically for appropriate time-delay if the network is connected. However, as the order of the consensus increases, the effects of high-order dynamics of the agents emerge, and the consensus problems are much more complicated. 

In this paper, we extend earlier work and introduce scalable arbitrary high-order consensus algorithms with communication time-delays. Via frequency domain analysis, we investigate the stability switches and establish an explicit general connection between the arbitrary high-order consensus and the system parameters, including the Laplacian spectrum of the underlying network topology, the feedback gains of the algorithms, and communication constraints. The main contribution of this paper is to provide a theoretical and computational framework for analysis and synthesis of scalable arbitrary high-order consensus algorithms in presence of communication time-delays. 

An outline of this paper is as follows.  Section 2 deals with basic concepts and notational details used throughout the paper and introduces the arbitrary high-order consensus algorithms with communication time-delays. The main theoretical results are given in Section 3, while Section 4 offers numerical simulation instances, showing the effectiveness of the present method. Finally, concluding remarks are presented in Section 5. 

## 2. Background and Problem Statement

Consider *n* dynamic agents with *k*th-order dynamics described by

(1) $$\begin{array}{c} \;{\dot{x}}_{i}^{\left(0\right)}\left(t\right)=x_{i}^{\left(1\right)}\left(t\right),\\ \vdots \\ {\dot{x}}_{i}^{\left(k-2\right)}\left(t\right)=x_{i}^{\left(k-1\right)}\left(t\right),\\ {\dot{x}}_{i}^{\left(k-1\right)}\left(t\right)=u_{i}\left(t\right), \end{array}$$

where *i* = 1,2,…, *n*, *x*_*i*_^(*m*)^(*t*) ∈ *R*, *m* = 0,1,…, *k* − 1, and *u*_*i*_(*t*) ∈ *R* denote the information states and the control input of agent *i*, respectively. *x*_*i*_^(*m*)^(*t*) denotes the *m*th derivative of *x*_*i*_ with *x*_*i*_^(0)^(*t*) = *x*_*i*_(*t*). Define the states of the whole network as

(2) $$\begin{array}{c} X\left(t\right)={\left[(x^{\left(0\right)}{\left(t)\right)}^{T},\ldots ,(x^{\left(k-1\right)}{\left(t\right))}^{T}\right]}^{T}, \end{array}$$

where **x** ^(*m*)^(*t*) = [*x*_1_^(*m*)^(*t*),…, *x*_*n*_^(*m*)^(*t*)]^*T*^, *m* = 0,1,…, *k* − 1. Then, we can decompose **x** ^(*m*)^(*t*) according to the following equation:

(3) $$\begin{array}{c} x^{\left(m\right)}\left(t\right)={\overset{-}{x}}^{\left(m\right)}\left(t\right)1+\delta^{\left(m\right)}\left(t\right), \end{array}$$

where ${\overset{-}{x}}^{\left(m\right)}(t)=\sum_{i}x_{i}^{\left(m\right)}(t)/n$, 1 = [1,1,…, 1]^*T*^ ∈ **R** ^*n*^, and **δ** ^(*m*)^(*t*) ∈ **R** ^*n*^. We refer to *e*(*t*) = ∑_*m*=0_^*k*−1^||**δ** ^(*m*)^(*t*)||^2^ as the overall group disagreement for the *k*th-order consensus problems, where ||·|| denotes the Euclidean norm. The set of integers, real numbers, and complex numbers are denoted by **Z**,  **R**, and **C**, respectively.

Information exchange between agents can be naturally modeled by the weighted undirected graph *G* = (*V*, *E*, **A**), where *V* = {*v*_*i*_} is the set of agents, {*e*_*ij*_} = *E*⊆*V* × *V* is the set of links between the agents, and **A** is the corresponding weighted adjacency matrix. The adjacency matrix **A** = [*a*_*ij*_] ∈ *R* ^*n*×*n*^ is defined such that *a*_*ij*_ > 0 if (*v*_*j*_, *v*_*i*_) ∈ *E*, while *a*_*ij*_ = 0 if (*v*_*j*_, *v*_*i*_) ∉ *E*. Therefore, the *a*_*ij*_ allow our results to be valid and useful for weighted network, that is, much more general than 0-1 weighted network. Let matrix **L** = [*l*_*ij*_] be defined as *l*_*ii*_ = ∑_*j*≠*i*_ *a*_*ij*_ and *l*_*ij*_ = −*a*_*ij*_, where *i* ≠ *j*. Following algebraic graph theory, **L** is positive semidefinite and is called the Laplacian matrix. The set of neighbors of agent *i* is defined as *N*_*i*_ = {*v*_*j*_ : (*v*_*j*_, *v*_*i*_) ∈ *E*}. The degree of the node *v*_*i*_ ∈ *V* and the average of the degrees of the vertices adjacent to *v*_*i*_ are denoted by *d*_*i*_ = |*N*_*i*_| and *m*_*i*_, respectively. The graph *G* does not contain a loop, a link joining an agent to itself. Suppose that each agent exchanges the information with its neighboring agents after the communication time-delay *τ*_*ij*_ > 0 corresponding to the link *e*_*ij*_ ∈ *E*.

We employ the following scalable time-delayed high-order consensus algorithm:

(4) $$\begin{array}{c} u_{i}\left(t\right)=\sum_{j\in N_{i}}a_{ij}\sum_{m=0}^{k-1}\beta_{m}\left(x_{j}^{\left(m\right)}\left(t-\tau_{ij}\right)-x_{i}^{\left(m\right)}\left(t-\tau_{ij}\right)\right), \end{array}$$

where *β*_*m*_ are positive constants denoting the feedback gains of the algorithm. 

The high-order consensus problem discussed in this paper is defined exactly as follows.

**Definition 1 (*k*th-order consensus)**

For the network of *k*th-order integrator systems, consensus is said to be reached globally asymptotically among dynamic agents if |*x*_*i*_^(*m*)^(*t*) − *x*_*j*_^(*m*)^(*t*)| → 0,  *m* = 0,1,…, *k* − 1, for all *i* ≠ *j* as *t* → *∞* for any *x*(0).

## 3. Main Results

### 3.1. General Stability Switches Criterion

In this section, we consider the high-order consensus problems in weighted networks of dynamic agents with *k*th-order integrator dynamics when the interaction is affected by communication time-delays. The following theorem provides the general formalism for the high-order consensus with communication time-delays and gives insight in the relation between the stability switches of general high-order consensus and the system parameters such as the network topology, communication time-delays, and feedback gains.

**Theorem 2**

Consider a network of n dynamic agents with dynamics described by (1). Assume that the network *G* = (*V*, *E*, **A**) is connected and that each agent receives the information from its neighboring agents after a constant time-delay *τ* > 0 and applies the control law (4). Then, the following statements are true:

- if all *K*_*i*_(*ω*): = *ω* ^2*k*^ − *μ*_*i*_^2^|∑_*m*=0_^*k*−1^ *β*_*m*_(*jω*)^*m*^|^2^ = 0 (2 ≤ *i* ≤ *n*) have no positive roots, then no stability switch of consensus may occur, and hence, if consensus is stable at *τ* = 0 it remains stable for all *τ* > 0, whereas if it is unstable at *τ* = 0 it remains unstable for all *τ* > 0;
- if there exist equations *K*_*i*_(*ω*) = 0 that have at least one positive root and each of them is simple, then as *τ* increases, a finite number of stability switches of consensus may occur, and eventually the consensus becomes unstable.

**Proof**

Since the graph *G* is connected, the Laplacian matrix **L** of *G* has a simple zero eigenvalue, and all the other eigenvalues are positive real numbers. Hence, −**L** has exactly one zero eigenvalue, and all the other eigenvalues are negative real numbers. Therefore, we write the eigenvalues of −**L** in the form *μ*_*n*_ ≤ *μ*_*n*−1_ ≤ ⋯≤*μ*_2_ < *μ*_1_ = 0. Given the time-delayed high-order algorithm (4), the network dynamics can be written as

(5) $$\begin{array}{c} {\dot{x}}^{\left(0\right)}\left(t\right)=x^{\left(1\right)}\left(t\right),\\ \vdots \\ {\dot{x}}^{\left(k-2\right)}\left(t\right)=x^{\left(k-1\right)}\left(t\right),\\ {\dot{x}}^{\left(k-1\right)}\left(t\right)=-L\sum_{m=0}^{k-1}\beta_{m}x^{\left(m\right)}\left(t-\tau \right). \end{array}$$

Despite the nonzero time-delay *τ* existing in the network, we still have that ${\overset{-}{x}}^{\left(k-1\right)}$ is an invariant quality during the transient process. Then, by employing the appropriate linear transformations **z** ^(*m*)^(*t*) = **U** ^*T*^ **x** ^(*m*)^(*t*), the closed-loop dynamics equations can be decoupled into *n* noninteracting subsystems

(6) $$\begin{array}{c} {\dot{z}}_{i}^{\left(0\right)}\left(t\right)=z_{i}^{\left(1\right)}\left(t\right),\\ \vdots \\ {\dot{z}}_{i}^{\left(k-2\right)}\left(t\right)=z_{i}^{\left(k-1\right)}\left(t\right),\\ {\dot{z}}_{i}^{\left(k-1\right)}\left(t\right)=\mu_{i}\sum_{m=0}^{k-1}\beta_{m}{\dot{z}}_{i}^{\left(m\right)}\left(t-\tau \right), \end{array}$$

where *i* = 1,2,…, *n* and *z*_*i*_^(*m*)^(*t*) is the *i*th components of **z** ^(*m*)^(*t*). In order to establish the stability of the high-order consensus system, our proof heavily depends on the frequency domain analysis. After taking the Laplace transform of the last set of equations, we get

(7) $$\begin{array}{c} \left[\begin{array}{c} z_{i}^{\left(0\right)}\left(s\right)\\ \vdots \\ z_{i}^{\left(k-2\right)}\left(s\right)\\ z_{i}^{\left(k-1\right)}\left(s\right) \end{array}\right]={\left(sI_{k}-\Omega_{i}\left(s\right)\right)}^{-1}\left[\begin{array}{c} z_{i}^{\left(0\right)}\left(0\right)\\ \vdots \\ z_{i}^{\left(k-2\right)}\left(0\right)\\ z_{i}^{\left(k-1\right)}\left(0\right) \end{array}\right], \end{array}$$

where

(8) $$\begin{array}{c} \Omega_{i}\left(s\right)=\left[\begin{array}{cccc} 0 & 1 & \cdots & 0\\ \vdots & \vdots & \ddots & \vdots \\ 0 & 0 & \cdots & 1\\ \beta_{0}\mu_{i}e^{-\tau s} & \cdots & \beta_{k-2}\mu_{i}e^{-\tau s} & \beta_{k-1}\mu_{i}e^{-\tau s} \end{array}\right], \end{array}$$

*s* is the Laplace variable, and **I**_*k*_ is the *k* × *k* identity matrix.

Denote **Ξ**_*i*_(*s*) = *s* **I**_*k*_ − **Ω**_*i*_(*s*). Let (*s*, [*f*_*i*0_,…, *f*_*i*(*k*−2)_, *f*_*i*(*k*−1)_]^*T*^) be a right MIMO transmission zero of **Ξ**_*i*_(*s*) at frequency *s* in the direction [*f*_*i*0_,…, *f*_*i*(*k*−2)_, *f*_*i*(*k*−1)_]^*T*^; that is, **Ξ**_*i*_(*s*)[*f*_*i*0_,…, *f*_*i*(*k*−2)_, *f*_*i*(*k*−1)_]^*T*^ = 0, where *s* ∈ **C** and [*f*_*i*0_,…, *f*_*i*(*k*−2)_, *f*_*i*(*k*−1)_]^*T*^ ≠ 0. Then, we find that

(9) $$\begin{array}{c} sf_{i0}-f_{i1}=0,\\ \vdots \\ sf_{i(k-2)}-f_{i(k-1)}=0,\\ -e^{-\tau s}\mu_{i}\sum_{m=0}^{k-1}\beta_{m}f_{im}+sf_{i(k-1)}=0, \end{array}$$

and therefore,

(10) $$\begin{array}{c} \left[s^{k}-e^{-\tau s}\mu_{i}\sum_{m=0}^{k-1}\beta_{m}s^{m}\right]f_{i0}=0. \end{array}$$

It is obvious that *f*_*i*0_ ≠ 0. Thus, (10) tells us that the poles of the *i*th subsystem described by (6) can be determined according to the following fundamental transcendental equations:

(11) $$\begin{array}{c} Q_{i}\left(s\right):=s^{k}-e^{-\tau s}\mu_{i}\sum_{m=0}^{k-1}\beta_{m}s^{m}=0, \end{array}$$

where *i* = 1,2,…, *n*.

From (6), we note that the first subsystem (for *μ*_1_ = 0) is marginally stable. Hence, for the high-order consensus to be stable, all the other subsystems (for *μ*_*i*_(2 ≤ *i* ≤ *n*)) have to be asymptotically stable, which means that all the poles (i.e., roots) given by (11), for 2 ≤ *i* ≤ *n*, need to be located in the open left half-plane (LHP). Therefore, it is sufficient to consider the location of the roots of the equations *Q*_*i*_(*s*) = 0 for 2 ≤ *i* ≤ *n*. Since

(12) lim⁡sup⁡Re(s)>0|s|→∞|s−kμi∑m=0k−1βmsm|<1,

we conclude that the total multiplicity *D*_*i*_(*τ*) of roots of *Q*_*i*_(*s*) = 0 in the open right half-plane is finite, and *D*_*i*_(*τ*) can change only if a root appears on or crosses the imaginary axis.

As communication time-delay *τ* increases, it may happen that roots cross the imaginary axis, and the equations *Q*_*i*_(*s*) = 0 may change from stable to unstable or vice versa, and accordingly, the stability of consensus may switch. If so, we say that there has been a stability switch of consensus. Now, we examine the location of roots and the direction of motion as they cross the imaginary axis as follows.

Assume that *s* = *jω* ≠ 0 is a root of (11). Because *M*(*s*): = *s* ^*k*^ and *N*_*i*_(*s*): = −*μ*_*i*_∑_*m*=0_^*k*−1^ *β*_*m*_ *s* ^*m*^ (2 ≤ *i* ≤ *n*) are polynomials of real coefficients, we may choose *ω* > 0 without loss of generality. Equation (11) implies that *K*_*i*_(*ω*) = 0, which clearly implies (a) of our theorem.

Next, assume there exist equations *K*_*i*_(*ω*) = 0 (*i* ∈ Δ⊆{2,3,…, *n*}) that have at least one positive root and each of them is simple. Our discussion thereafter is limited to such nonempty set *i* ∈ Δ. We set *M*(*jω*) = *M*_*R*_(*ω*) + *jM*_*I*_(*ω*) and *N*_*i*_(*jω*) = *N*_*iR*_(*ω*) + *jN*_*iI*_(*ω*), where *M*_*R*_, *M*_*I*_, *N*_*iR*_, and *N*_*iI*_ are real-valued functions. Then, we find that (11) holds if and only if

(13) $$\begin{array}{c} N_{iR}\cos\left(\omega \tau \right)+N_{iI}\sin\left(\omega \tau \right)=-M_{R},\\ N_{iI}\cos\left(\omega \tau \right)-N_{iR}\sin\left(\omega \tau \right)=-M_{I}. \end{array}$$

Therefore,

(14) $$\begin{array}{c} \sin\left(\omega \tau \right)=\frac{-M_{R}N_{iI}+M_{I}N_{iR}}{{\left|N_{i}\right|}^{2}}, \end{array}$$

(15) $$\begin{array}{c} \cos\left(\omega \tau \right)=\frac{-M_{R}N_{iR}-M_{I}N_{iI}}{{\left|N_{i}\right|}^{2}}, \end{array}$$

where 0 ≤ *ωτ* < 2*π*. For each root of *K*_*i*_(*ω*) = 0(*i* ∈ Δ), it may be possible to calculate all values of *τ* > 0 that satisfy (14) and (15).

Now suppose that we have obtained values of *ω*, *τ* by the previous procedure. We regard the root *s*(*τ*) of (11) as a function of time-delay *τ*, and we need to determine the direction of motion of *Re*(*s*(*τ*)) as *τ* is varied. That is, we calculate

(16) Φ=sign⁡(ddτRe(s(τ))|s=jω)=sign⁡(Re(ddτs(τ)|s=jω)).

Since *Q*_*i*_(*s*) is an analytic function of *s* and *τ*, a root *s*(*τ*) will be a differentiable function of time-delay *τ*, except at points where the root is multiple.

Then, differentiating (11) with respect to *τ* gives

(17) $$\begin{array}{c} {\left(\frac{\text{d}s}{\text{d}\tau }\right)}^{-1}=-\frac{M^{\prime }\left(s\right)}{sM\left(s\right)}+\frac{N_{i}^{\prime }\left(s\right)}{sN_{i}\left(s\right)}-\frac{\tau }{s}, \end{array}$$

which holds at any simple root *jω* of (11) with *i* ∈ Δ. Therefore,

(18) Φ=sign(Re(ddτs(τ)|s=jω))=sign⁡Re(−M′(jω)jωM(jω)+Ni′(jω)jωNi(jω)−τjω)=sign⁡Re(−M′(jω)jωM(jω)+Ni′(jω)jωNi(jω))=−sign⁡Im⁡(M′(jω)M(jω)−Ni′(jω)Ni(jω))=sign⁡[−Im⁡(M′(jω)M−(jω)−Ni′(jω)N−i(jω))]=sign⁡[MRMR′+MIMI′−NiRNiR′−NiINiI′]=sign[Ki′(ω)]=sign[ddω(ω2k−μi2|∑m=0k−1βm(jω)m|2)].

The last line in (18) is a central formula that explicitly connects the horizontal moving sense of a root of (11) when communication time-delay *τ* increases with the properties of the network topology and feedback gains.

Suppose that *ω*_*i*1_ > *ω*_*i*2_ > ⋯>*ω*_*ip*_ > 0 are constants such that *jω*_*id*_, *d* = 1,2,…, *p*, are simple roots of our *K*_*i*_(*ω*) = 0 corresponding to *μ*_*i*_ (*i* ∈ Δ) that cross the imaginary axis at *jω*_*id*_ at time-delay values *τ*_*idr*_ (*r* = 1,2,…) determined by (14) and (15). At each crossing, the number of roots in the half-plane *Re*(*s*) > 0 changes by two, as roots occur in conjugate pairs. Since *K*_*i*_′(*ω*_*id*_) and *K*_*i*_′(*ω*_*i*(*d*+1)_) have opposite signs, we observe that crossing at two adjacent simple roots *jω*_*id*_ and *jω*_*i*(*d*+1)_ must be in opposite moving directions. Moreover, for crossing at a given root *jω*_*id*_, the difference between the adjacent time-delay values is *τ*_*id*(*r*+1)_ − *τ*_*idr*_ = 2*π*/*ω*_*id*_. Hence, on the average, crossings occur most frequently at *jω*_*i*1_, next most frequently at *jω*_*i*2_,…, and least often at *jω*_*ip*_, which implies that crossings at *jω*_*i*(2*q*+1)_ must be to the right and crossings at *jω*_*i*(2*q*)_ must be to the left. Then, as *τ* increases, a finite number of stability switches of consensus may occur, and eventually the consensus becomes unstable. This clearly implies (b) of our theorem.

### 3.2. Analytical Results on the Maximum Tolerable Communication Time-Delays for High-Order Consensus

Theorem 2 provides a fairly general and precise notion of how increasing communication time-delay affects the stability of arbitrary high-order consensus and sheds light on the relation between the graph Laplacian spectrum of the underlying network and the convergence properties of the proposed consensus algorithm. Furthermore, the method of proof given develops a way to determine the critical values of time-delay *τ* where stability switch of consensus may occur. With Theorem 2 in hand, we may even derive closed-form analytical results on the maximum tolerable communication time-delays for consensus problems with specific orders. 

We now introduce the following existing results reported in Olfati-Saber and Murray [5] for the first-order consensus case and our earlier work [16] for the second-order case, respectively. It can be shown that these two statements are routine corollaries of Theorem 2 which we derived in this paper.

**Corollary 3 (see [5])**

Consider a network of first-order integrator agents. Assume that the network *G* = (*V*, *E*, **A**) is connected and that each agent receives the information from its neighboring agents after a constant time-delay *τ* > 0 and applies the control law (4). Then, the network solves first-order consensus if and only if *τ* ∈ [0, τ*) with

(19) $$\begin{array}{c} \tau^{*}=-\frac{\pi }{2\beta_{0}\mu_{n}}. \end{array}$$

**Corollary 4 (see [16])**

Consider a network of second-order integrator agents. Assume that the network *G* = (*V*, *E*, **A**) is connected and that each agent receives the information from its neighboring agents after a constant time-delay *τ* > 0 and applies the control law (4). Then, the network solves second-order consensus if and only if *τ* ∈ [0, τ*) with

(20) $$\begin{array}{c} \tau^{*}=\underset{2\leq i\leq n}{\min}\left\{\arctan\frac{\left(\beta_{1}\omega_{i}/\beta_{0}\right)}{\omega_{i}}\right\}, \end{array}$$

where

(21) $$\begin{array}{c} \omega_{i}={\left(\frac{(\mu_{i}^{2}\beta_{1}^{2}+\left(\mu_{i}^{4}\beta_{1}^{4}{+4\mu_{i}^{2}\beta_{0}^{2})}^{1/2}\right)}{2}\right)}^{1/2}. \end{array}$$

Now we give the necessary and sufficient condition for the stability of the third-order consensus in multiagent networks with time-delays, with the purpose of showing how Theorem 2 can be applied to consensus problems with specific orders.

**Corollary 5**

Consider a network of third-order integrator agents. Assume that the network *G* = (*V*, *E*, **A**) is connected and that each agent receives the information from its neighboring agents after a constant time-delay *τ* > 0 and applies the control law (4). Suppose that the network topology and feedback gains satisfy the following conditions:

(22)  (i)  μ2<−β0β1β2;(ii)  β12−2β0β2>0.

Then, the network solves third-order consensus if and only if *τ* ∈ [0, τ*) with

(23) $$\begin{array}{c} \tau^{*}=\underset{2\leq i\leq n}{\min}\left\{\frac{\theta_{i}}{\omega_{i}}\right\}, \end{array}$$

*where ω*_*i*_ *and θ*_*i*_ *are given by *(24), (25),* and *(26).

**Proof**

For the third-order consensus, we have *k* = 3, *M*(*s*) = *s* ^3^, and *N*_*i*_(*s*) = −*μ*_*i*_∑_*m*=0_^2^ *β*_*m*_ *s* ^*m*^. When *τ* = 0, (11) degenerates to the polynomials *s* ^3^ − *μ*_*i*_ *β*_2_ *s* ^2^ − *μ*_*i*_ *β*_1_ *s* − *μ*_*i*_ *β*_0_ = 0, which are Hurwitz stable for the nonidentically zero eigenvalues *μ*_*i*_ (2 ≤ *i* ≤ *n*) if and only if hypothesis (i) holds. When *τ* > 0, the equation

(24) $$\begin{array}{c} K_{i}\left(\omega \right)=\omega^{6}-\mu_{i}^{2}\beta_{2}^{2}\omega^{4}-\mu_{i}^{2}\left(\beta_{1}^{2}-2\beta_{0}\beta_{2}\right)\omega^{2}-\mu_{i}^{2}\beta_{0}^{2}=0 \end{array}$$

is a cubic equation in *ω* ^2^, and hence, the cubic formula can be applied to yield the closed-form solution. Furthermore, by Descartes' Rule of Signs, we see that the cubic equation in *ω* ^2^ has exactly one positive real root for all 2 ≤ *i* ≤ *n* due to hypothesis (ii). Therefore, the existence and uniqueness of positive real roots *ω*_*i*_ > 0 of the original (24) are guaranteed. Then, the crossing at *jω*_*i*_ must be to the right. Using (14) and (15), the critical communication time-delay *τ* can be given by

(25) $$\begin{array}{c} \sin\left(\theta \right)=\frac{\omega^{3}\left(\beta_{0}-\beta_{2}\omega^{2}\right)}{\mu_{i}\left[{\left(\beta_{0}-\beta_{2}\omega^{2}\right)}^{2}+\beta_{1}^{2}\omega^{2}\right]}, \end{array}$$

(26) $$\begin{array}{c} \cos\left(\theta \right)=\frac{-\omega^{3}\beta_{1}\omega }{\mu_{i}\left[{\left(\beta_{0}-\beta_{2}\omega^{2}\right)}^{2}+\beta_{1}^{2}\omega^{2}\right]}, \end{array}$$

where 0 ≤ *θ* < 2*π*. Then, we have the following set of values of *τ*_*ir*_ corresponding to *μ*_*i*_ for which there are imaginary roots:

(27) $$\begin{array}{c} \tau_{ir}=\frac{\theta_{i}}{\omega_{i}}+\frac{2r\pi }{\omega_{i}}, \end{array}$$

where *r* = 0,1, 2,….

Hence, the explicit expression for the tight upper bound on the time-delays is given by τ* = min⁡_2≤*i*≤*n*_{*θ*_*i*_/*ω*_*i*_}. Then we conclude from Theorem 2 that when 0 ≤ *τ* < τ*, the consensus is stable and when *τ* > τ*, it is unstable. Moreover, the system has a globally asymptotically stable oscillatory solution when *τ* = τ*.

**Remark 6**

The maximum tolerable communication time-delay is a fundamental performance measure for consensus and hence plays an important role in the design of distributed coordination of multiagent systems. It is shown previously that the communication constraints affect the stability of a high-order consensus process in a rather sophisticated fashion. Our results imply that the optimal (or suboptimal) feedback gains and network topologies can be synthesized such that the general high-order consensus robustness of the dynamic network to the communication time-delays is maximized. Thus, in that sense our results can shed light on the whole distributed cooperative control design.

## 4. Numerical Example and Simulation Results

**Figure 1:**
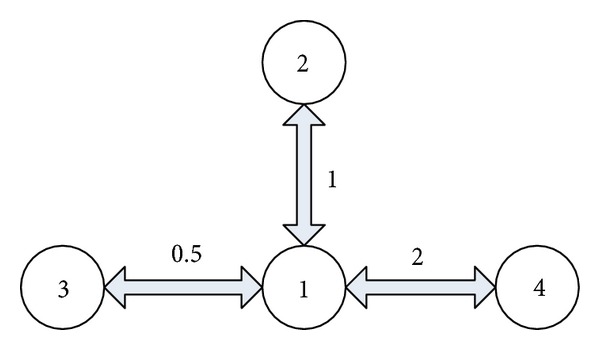
Undirected communication graph *G* used for fourth-order consensus problem.

We stress that the results in this paper characterize the robustness of the distributed algorithms to the communication time-delays existing in the network for the arbitrary high-order consensus. In order to illustrate this point, we consider solving fourth-order consensus problem of the network of four agents, whose communication graph *G* is shown in Figure 1. It is easy to see that *G* is a connected graph. We assume that the numbers on the links are the corresponding weights of the communication links in the graph *G*, and that the communication time-delay *τ* ≥ 0 is time invariant. Moreover, the agents evolve according to (1) and (4) starting from a random initial conditions that can be arbitrary continuous function defined on [−*τ*, 0]. We assume that the communication time-delay *τ* ≥ 0 is time invariant. The algorithm parameters are assigned to the values *β*_0_ = 1, *β*_1_ = 1, *β*_2_ = 3, and *β*_3_ = 1. By Theorem 2, we have *k* = 4, *M*(*s*) = *s* ^4^, and *N*_*i*_(*s*) = −*μ*_*i*_∑_*m*=0_^3^ *β*_*m*_ *s* ^*m*^. Then all equations *K*_*i*_(*ω*) = 0, 2 ≤ *i* ≤ 4 have exactly one positive real root for all *ω*_2_ = 1.1760, *ω*_3_ = 2.0311, and *ω*_4_ = 5.6174. Thus, the corresponding crossings at *jω*_*i*_ for all *Q*_*i*_(*s*) = 0 must be to the right. Furthermore, when *τ* = 0, the consensus is stable, since the characteristic polynomials

(28) $$\begin{array}{c} P_{i}\left(s\right)=s^{4}-\mu_{i}\beta_{3}s^{3}-\mu_{i}\beta_{2}s^{2}-\mu_{i}\beta_{1}s-\mu_{i}\beta_{0} \end{array}$$

are Hurwitz stable for the nonidentically zero eigenvalues *μ*_*i*_. Hence, as communication time-delay *τ* increases, exactly one stability switch of consensus can occur, and the consensus becomes unstable after such switch. Using (14) and (15), the switch point (i.e., the tight upper bound on the time-delays) is given by τ* ≈ 0.1208. 

**Figure 2:**
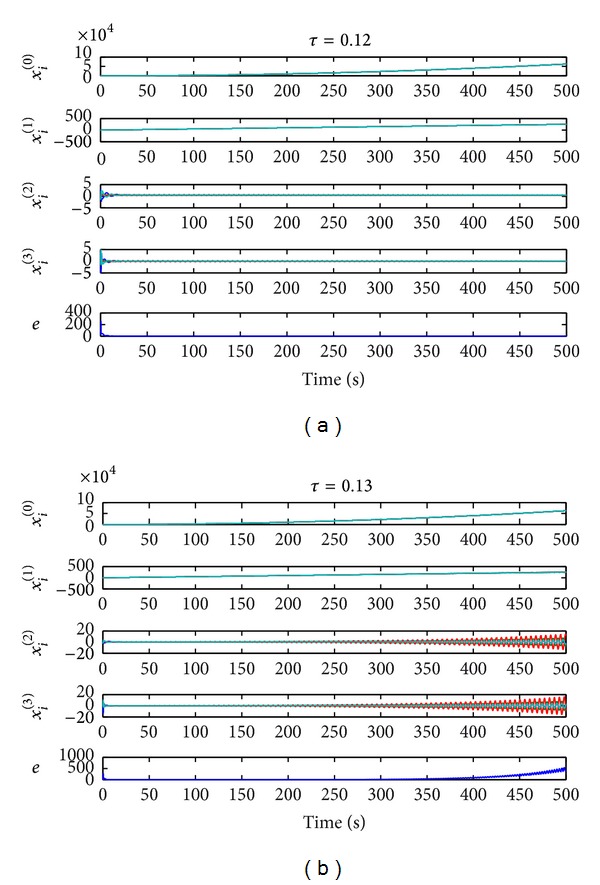
Evolutions of the fourth-order consensus with communication time-delays on graph *G* given in Figure 1: (a) *τ* = 0.12 and (b) *τ* = 0.13.

In the first simulation experiment, we choose the time-delay *τ* = 0.12 that is slightly below τ*. Then, Theorem 2 guarantees fourth-order consensus, and such a result can be clearly verified from Figure 2(a), where the evolution of fourth-order consensus system is represented. In the second simulation experiment, we use *τ* = 0.13, so having *τ* > τ*. As predicted by our theory, the dynamics becomes unstable, and fourth-order consensus cannot be achieved. This can be seen in Figure 2(b). The numerical simulation results are consistent with the theoretical results.

## 5. Conclusions 

This paper has presented arbitrary high-order consensus algorithms for information consensus in networks of dynamic agents suffering from communication time-delays. Using frequency domain method, a rigorous and general convergence analysis was given. By taking communication time-delay *τ* as a parameter and examining the location of poles and direction of motion as they cross the imaginary axis, we established our central result of arbitrary high-order consensus, Theorem 2. In addition, we developed a systematic method to determine the critical value(s) of communication time-delay *τ* at which stability switches of consensus (if any) occur. The sufficient and necessary conditions for the stability of the consensus up to third-order under mild assumptions were given, with the purpose of showing how Theorem 2 can be applied to consensus problems with specific orders. Numerical simulation results were provided to demonstrate the effectiveness of our theoretical results and analytical tools. We suggest that insights provided by these results will illuminate the design principles and evolution mechanisms of both natural and engineered dynamic network, where consensus is functionally significant.

## References

[1] Leonard NE, Shen T, Nabet B, Scardovi L, Couzin ID, Levin SA (2012). Decision versus compromise for animal groups in motion. *Proceedings of the National Academy of Sciences of the United States of America*.

[2] Pillot MH, Gautrais J, Arrufat P, Couzin ID, Bon R, Deneubourg JL (2011). Scalable rules for coherent group motion in a gregarious vertebrate. *PLoS ONE*.

[3] Jadbabaie A, Lin J, Morse AS (2003). Coordination of groups of mobile autonomous agents using nearest neighbor rules. *IEEE Transactions on Automatic Control*.

[4] Moreau L (2005). Stability of multiagent systems with time-dependent communication links. *IEEE Transactions on Automatic Control*.

[5] Olfati-Saber R, Murray RM (2004). Consensus problems in networks of agents with switching topology and time-delays. *IEEE Transactions on Automatic Control*.

[6] Ren W (2007). Consensus strategies for cooperative control of vehicle formations. *IET Control Theory and Applications*.

[7] Ren W, Beard RW (2005). Consensus seeking in multiagent systems under dynamically changing interaction topologies. *IEEE Transactions on Automatic Control*.

[8] Ren W, Beard RW, Atkins EM (2007). Information consensus in multivehicle cooperative control. *IEEE Control Systems Magazine*.

[9] Bauso D, Giarré L, Pesenti R (2006). Non-linear protocols for optimal distributed consensus in networks of dynamic agents. *Systems and Control Letters*.

[10] Olfati-Saber R, Fax JA, Murray RM (2007). Consensus and cooperation in networked multi-agent systems. *Proceedings of the IEEE*.

[11] Porfiri M, Stilwell DL (2007). Consensus seeking over random weighted directed graphs. *IEEE Transactions on Automatic Control*.

[12] Savkin AV (2006). The problem of coordination and consensus achievement in groups of autonomous mobile robots with limited communication. *Nonlinear Analysis, Theory, Methods and Applications*.

[13] Bliman PA, Ferrari-Trecate G (2008). Average consensus problems in networks of agents with delayed communications. *Automatica*.

[14] Yu J, Wang L (2010). Group consensus in multi-agent systems with switching topologies and communication delays. *Systems and Control Letters*.

[15] Lin P, Jia Y (2011). Multi-agent consensus with diverse time-delays and jointly-connected topologies. *Automatica*.

[16] Yang B, Fang H (2010). Forced consensus in networks of double integrator systems with delayed input. *Automatica*.

[17] Gazi V, Passino KM (2003). Stability analysis of swarms. *IEEE Transactions on Automatic Control*.

[18] Gazi V, Passino KM (2004). Stability analysis of social foraging swarms. *IEEE Transactions on Systems, Man, and Cybernetics B*.

[19] Tanner HG, Christodoulakis DK (2007). Decentralized cooperative control of heterogeneous vehicle groups. *Robotics and Autonomous Systems*.

[20] Tanner HG, Jadbabaie A, Pappas GJ (2007). Flocking in fixed and switching networks. *IEEE Transactions on Automatic Control*.

[21] Slotine JJE, Li W (1991). *Applied Nonlinear Control*.

[22] Couzin ID, Krause J, Slater PJB, Rosenblatt JS, Snowdon CT, Roper TJ (2003). Self-organization and collective behavior in vertebrates. *Advances in the Study of Behavior*.

[23] Hale JK (1977). *Theory of Functional Differential Equations*.

